# Supplementary material for: Modeling glioblastoma heterogeneity as a dynamic network of cell states
Source: Mol Syst Biol. 2021 Sep 16;17(9):e10105. doi: 10.15252/msb.202010105 (PMC8444284; doi:10.15252/msb.202010105)
Supplement: Supplementary file 5 — Source Data for Figure 3 [file MSB-17-e10105-s001.zip › Figure3A_sourcedata/GSEA_3065/nosigdb_state2.GseaPreranked.1622718419215/RICHARDS_VERHAAK_CANCERCELL_2010_MESENCHYMAL.html]

Details for gene set RICHARDS\_VERHAAK\_CANCERCELL\_2010\_MESENCHYMAL[GSEA]

|  || Dataset | state2 |
| Phenotype | NoPhenotypeAvailable |
| Upregulated in class | na\_neg |
| GeneSet | RICHARDS\_VERHAAK\_CANCERCELL\_2010\_MESENCHYMAL |
| Enrichment Score (ES) | -0.65499127 |
| Normalized Enrichment Score (NES) | -2.5407379 |
| Nominal p-value | 0.0 |
| FDR q-value | 0.0 |
| FWER p-Value | 0.0 |
Table: GSEA Results Summary

  

Fig 1: Enrichment plot: RICHARDS\_VERHAAK\_CANCERCELL\_2010\_MESENCHYMAL      
 Profile of the Running ES Score & Positions of GeneSet Members on the Rank Ordered List

  

| PROBE | GENE SYMBOL | GENE\_TITLE | RANK IN GENE LIST | RANK METRIC SCORE | RUNNING ES | CORE ENRICHMENT || 1 | CLIC1 |  |  | 1291 | 0.099 | -0.1269 | No |
| 2 | UCP2 |  |  | 1329 | 0.096 | -0.1198 | No |
| 3 | UAP1 |  |  | 1401 | 0.092 | -0.1168 | No |
| 4 | RHOG |  |  | 1944 | 0.067 | -0.1672 | No |
| 5 | RAB32 |  |  | 2054 | 0.063 | -0.1717 | No |
| 6 | TNFAIP8 |  |  | 2308 | 0.053 | -0.1926 | No |
| 7 | FCGR2A |  |  | 3770 | 0.019 | -0.3467 | No |
| 8 | WIPF1 |  |  | 3879 | 0.018 | -0.3563 | No |
| 9 | SLC10A3 |  |  | 4168 | 0.013 | -0.3856 | No |
| 10 | SH2B3 |  |  | 4182 | 0.012 | -0.3856 | No |
| 11 | YAP1 |  |  | 4184 | 0.012 | -0.3843 | No |
| 12 | S100A4 |  |  | 4483 | 0.008 | -0.4153 | No |
| 13 | CD2AP |  |  | 4604 | 0.006 | -0.4275 | No |
| 14 | PYGL |  |  | 4875 | 0.001 | -0.4562 | No |
| 15 | SWAP70 |  |  | 5075 | -0.001 | -0.4774 | No |
| 16 | SP100 |  |  | 5330 | -0.005 | -0.5040 | No |
| 17 | MGAT1 |  |  | 5429 | -0.007 | -0.5136 | No |
| 18 | ASL |  |  | 5484 | -0.008 | -0.5184 | No |
| 19 | CEBPB |  |  | 5541 | -0.009 | -0.5234 | No |
| 20 | S100A11 |  |  | 5569 | -0.009 | -0.5252 | No |
| 21 | DRAM1 |  |  | 5714 | -0.012 | -0.5393 | No |
| 22 | RABGAP1L |  |  | 5747 | -0.012 | -0.5413 | No |
| 23 | SFT2D2 |  |  | 5756 | -0.012 | -0.5407 | No |
| 24 | PDPN |  |  | 6446 | -0.024 | -0.6117 | No |
| 25 | TRADD |  |  | 6459 | -0.025 | -0.6101 | No |
| 26 | TMBIM1 |  |  | 6491 | -0.025 | -0.6106 | No |
| 27 | FURIN |  |  | 6544 | -0.026 | -0.6131 | No |
| 28 | PLA2G15 |  |  | 6548 | -0.026 | -0.6105 | No |
| 29 | PLK3 |  |  | 6607 | -0.027 | -0.6135 | No |
| 30 | VAMP5 |  |  | 6835 | -0.032 | -0.6341 | No |
| 31 | EFEMP2 |  |  | 6895 | -0.034 | -0.6365 | No |
| 32 | PLAUR |  |  | 6906 | -0.034 | -0.6337 | No |
| 33 | BLVRB |  |  | 7000 | -0.036 | -0.6394 | No |
| 34 | C1orf54 |  |  | 7100 | -0.039 | -0.6456 | No |
| 35 | RUNX2 |  |  | 7111 | -0.039 | -0.6421 | No |
| 36 | LHFPL2 |  |  | 7179 | -0.041 | -0.6446 | No |
| 37 | LRRFIP1 |  |  | 7193 | -0.042 | -0.6412 | No |
| 38 | DSE |  |  | 7207 | -0.042 | -0.6377 | No |
| 39 | EHD2 |  |  | 7220 | -0.043 | -0.6341 | No |
| 40 | ANXA1 |  |  | 7265 | -0.044 | -0.6338 | No |
| 41 | ZNF217 |  |  | 7333 | -0.045 | -0.6358 | No |
| 42 | ACSL1 |  |  | 7456 | -0.049 | -0.6432 | No |
| 43 | CPQ |  |  | 7468 | -0.050 | -0.6387 | No |
| 44 | HEXB |  |  | 7621 | -0.054 | -0.6488 | Yes |
| 45 | SYNGR2 |  |  | 7633 | -0.054 | -0.6438 | Yes |
| 46 | RBMS1 |  |  | 7667 | -0.055 | -0.6410 | Yes |
| 47 | CNN2 |  |  | 7676 | -0.055 | -0.6355 | Yes |
| 48 | ARPC1B |  |  | 7700 | -0.056 | -0.6315 | Yes |
| 49 | CYBRD1 |  |  | 7725 | -0.057 | -0.6275 | Yes |
| 50 | RRAS |  |  | 7768 | -0.059 | -0.6253 | Yes |
| 51 | COLGALT1 |  |  | 7959 | -0.065 | -0.6382 | Yes |
| 52 | MFSD1 |  |  | 8058 | -0.069 | -0.6407 | Yes |
| 53 | SEC24D |  |  | 8096 | -0.071 | -0.6365 | Yes |
| 54 | PLAU |  |  | 8131 | -0.073 | -0.6317 | Yes |
| 55 | TRIM22 |  |  | 8154 | -0.075 | -0.6255 | Yes |
| 56 | ITGA5 |  |  | 8155 | -0.075 | -0.6170 | Yes |
| 57 | ANXA4 |  |  | 8188 | -0.077 | -0.6116 | Yes |
| 58 | PIGP |  |  | 8274 | -0.081 | -0.6114 | Yes |
| 59 | NPC2 |  |  | 8297 | -0.083 | -0.6043 | Yes |
| 60 | LY96 |  |  | 8332 | -0.085 | -0.5983 | Yes |
| 61 | SHC1 |  |  | 8385 | -0.088 | -0.5937 | Yes |
| 62 | MVP |  |  | 8452 | -0.093 | -0.5902 | Yes |
| 63 | ARHGAP29 |  |  | 8476 | -0.094 | -0.5819 | Yes |
| 64 | MAN2A1 |  |  | 8487 | -0.095 | -0.5721 | Yes |
| 65 | SCPEP1 |  |  | 8654 | -0.107 | -0.5776 | Yes |
| 66 | LGALS1 |  |  | 8680 | -0.110 | -0.5677 | Yes |
| 67 | P4HA2 |  |  | 8700 | -0.111 | -0.5571 | Yes |
| 68 | WWTR1 |  |  | 8752 | -0.117 | -0.5491 | Yes |
| 69 | TGFBI |  |  | 8761 | -0.117 | -0.5365 | Yes |
| 70 | MYH9 |  |  | 8778 | -0.119 | -0.5246 | Yes |
| 71 | THBS1 |  |  | 8789 | -0.120 | -0.5119 | Yes |
| 72 | ADAM12 |  |  | 8799 | -0.122 | -0.4989 | Yes |
| 73 | MYOF |  |  | 8851 | -0.129 | -0.4895 | Yes |
| 74 | S100A13 |  |  | 8880 | -0.133 | -0.4773 | Yes |
| 75 | ANXA2 |  |  | 8891 | -0.134 | -0.4630 | Yes |
| 76 | FNDC3B |  |  | 8902 | -0.136 | -0.4485 | Yes |
| 77 | TGFBR2 |  |  | 8916 | -0.137 | -0.4342 | Yes |
| 78 | EMP3 |  |  | 8918 | -0.137 | -0.4186 | Yes |
| 79 | TNFRSF1A |  |  | 8962 | -0.143 | -0.4068 | Yes |
| 80 | IQGAP1 |  |  | 8965 | -0.143 | -0.3906 | Yes |
| 81 | ARSJ |  |  | 9083 | -0.163 | -0.3845 | Yes |
| 82 | TGOLN2 |  |  | 9105 | -0.167 | -0.3676 | Yes |
| 83 | MRC2 |  |  | 9106 | -0.167 | -0.3485 | Yes |
| 84 | HEXA |  |  | 9129 | -0.172 | -0.3311 | Yes |
| 85 | COL5A1 |  |  | 9148 | -0.177 | -0.3128 | Yes |
| 86 | CAST |  |  | 9164 | -0.179 | -0.2938 | Yes |
| 87 | SYPL1 |  |  | 9182 | -0.185 | -0.2745 | Yes |
| 88 | PLS3 |  |  | 9187 | -0.186 | -0.2536 | Yes |
| 89 | GRN |  |  | 9251 | -0.214 | -0.2358 | Yes |
| 90 | CTSB |  |  | 9255 | -0.215 | -0.2115 | Yes |
| 91 | SAT1 |  |  | 9279 | -0.224 | -0.1884 | Yes |
| 92 | LAMB1 |  |  | 9302 | -0.231 | -0.1643 | Yes |
| 93 | LGALS3 |  |  | 9308 | -0.236 | -0.1378 | Yes |
| 94 | TIMP1 |  |  | 9387 | -0.334 | -0.1079 | Yes |
| 95 | CTSC |  |  | 9413 | -0.431 | -0.0611 | Yes |
| 96 | DCBLD2 |  |  | 9431 | -0.558 | 0.0010 | Yes |
Table: GSEA details [plain text format]

  

Fig 2: RICHARDS\_VERHAAK\_CANCERCELL\_2010\_MESENCHYMAL: Random ES distribution      
 Gene set null distribution of ES for **RICHARDS\_VERHAAK\_CANCERCELL\_2010\_MESENCHYMAL**
